# Effects of induced relative hypoxia during the postoperative period of abdominal oncologic surgery, on hemoglobin and reticulocyte levels: a prospective, randomized controlled clinical trial

**DOI:** 10.1186/cc10869

**Published:** 2012-03-20

**Authors:** M Khalife, K Wiams, M Ben Aziz, M Paesmans, C Balestra, M Sosnowski

**Affiliations:** 1 Institut Jules Bordet, Brussels, Belgium; 2 Divert Alert Network Europe Research Division, Brussels, Belgium

## Introduction

Anemia is a frequent complication in oncologic patients. Erythropoietin (EPO) stimulating agents are known as alternatives to transfusion. However, they expose patients to thrombosis and are expensive. Recently, a new phenomenon, the normobaric oxygen paradox (NOP), has been described. In brief, transient hyperoxia followed by a prolonged return to normoxia acts as an effective trigger for EPO production. The mechanism depends on free oxygen radicals and on reduced glutathione (GSH) availabilities. Also, *N*-acetylcystein (NAC) is known to regenerate the stock of GSH. Very few clinical trials have investigated this phenomenon [1]. The goal of this study was to test the NOP theory on the evolution of hemoglobin and reticulocytes in patients receiving intermittent oxygen with or without NAC compared to a control group.

## Methods

This prospective, randomized study included 78 patients (three groups). The first group (G1; *n *= 26) received 60% FiO_2 _for 2 consecutive hours on the first, third, and fifth days postoperatively. The second group (G2; *n *= 26) in addition to oxygen received NAC 200 mg/day for 5 days. The third group (G3; *n *= 26) was the control group which did not receive any oxygen variation. On postoperative day 6, hemoglobin, hematocrit and reticulocytes were measured and compared to the baseline values. A total of five patients (three in G1 and two in G2) were excluded for discontinuing oxygen and/or early discharge from hospital.

## Results

**Figure 1:**
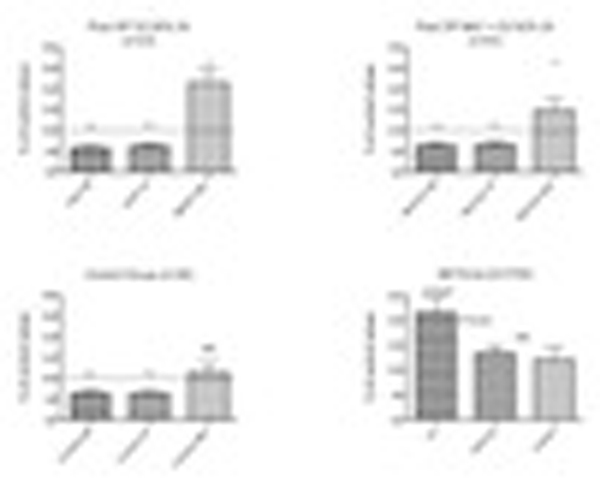


The reticulocyte count in G1 showed statistically different values compared to G2 and G3. These findings correlate with other clinical trials [2]. The fact that no statistical difference of hemoglobin level was recorded could be attributed to the lack of follow-up after patient discharge (postoperative day 6). See Figure 1.

## Conclusion

Induced relative hypoxia seems to be an effective stimulus for reticulocyte synthesis. However, further investigations are needed to confirm these findings and their impact on hemoglobin.
